# Identity Disturbance in the Digital Era during the COVID-19 Pandemic: The Adverse Effects of Social Media and Job Stress

**DOI:** 10.3390/bs14080648

**Published:** 2024-07-27

**Authors:** Bojan Obrenovic, Danijela Godinic, Gang Du, Akmal Khudaykulov, Hui Gan

**Affiliations:** 1 Zagreb School of Economics and Management, 10000 Zagreb, Croatia; bojan.obrenovic@zsem.hr; 2 School of Business and Management, Q University, Almaty 050026, Kazakhstan; 3 Faculty of Humanities and Social Sciences, University of Zagreb, 10000 Zagreb, Croatia; danijela.godinic5@gmail.com; 4 Social Sciences Department, Communication University of Zhejiang, Hangzhou 310018, China; 5 Trinity Business School, Trinity College Dublin, The University of Dublin, D02 F6N2 Dublin, Ireland; khudayka@tcd.ie; 6 Multimedia Department, Sungkyunkwan University, Seoul 03603, Republic of Korea; ganhui6688@outlook.com

**Keywords:** social media exposure, job stress, anxiety, identity disturbance, COVID-19 pandemic, mental health

## Abstract

The empirical study aimed to explore the relationships among social media exposure, job stress, anxiety, and identity disturbance in a nonclinical setting in the COVID-19 pandemic context. An online questionnaire was administered to 282 participants in the United States of America (USA) during the COVID-19 pandemic. The study utilized a two-step Structural Equation Modeling (SEM) approach consisting of both measurement model and structural model testing. Relationships between the model variables of social media exposure, identity disturbance, anxiety, and job stress were analyzed using standardized beta coefficients, standard errors, t-values, and *p*-values. The results indicate that both social media exposure and job stress are associated with increased anxiety levels, which, in turn, influence identity disturbance. Moreover, there is a moderating effect of job stress on the relationship between social media exposure and anxiety, as well as the mediating effect of anxiety on the relationship between social media exposure and identity disturbance. The findings are valuable for organizations and can be used to develop programs aimed at mitigating the adverse effects of social media exposure on mental health. Prioritizing employee mental health through awareness and support initiatives is paramount, especially for those facing high stress and extensive social media use.

## 1. Introduction

Coverage of the COVID-19 pandemic across digital media platforms has been widespread. Social media has been a key source of both accurate updates and significant misinformation [1]. This spread of false information on COVID-19 via social media has led to public misconceptions about increased health risks [2]. The overwhelming information and misinformation during the pandemic have fostered uncertainty and fear severely impacting mental health [3,4]. Furthermore, the digital era, characterized by increasing technological demands, has blurred the boundaries between the personal and work spheres [5]. This was made all the more evident during the pandemic, when employees were especially vulnerable to strain due to the remote working policy. They often had additional tasks received via digital channels outside working hours while executing family roles and managing their personal life [6]. An increased demand for availability is known to trigger anxiety and cause burnout and emotional distress [7]. By combining the prominent theoretical frameworks of the Job Demands–Resources (JD–R) model and boundary theory, this study examines the adverse psychological outcomes for individual employees faced with underlying anxiety and psychological strain under pressure from social media exposure during COVID-19 pandemic. The pandemic forced organizations to innovate, often needing to change their operational models and transition to a virtual environment [8]. Moreover, the lack of physical contact urged employers to appropriate the usage of networking platforms to maintain constant connectivity among team members. However, as was previously found [9], social media exposure has adverse effects on both the personal and professional domains, and it likely reinforces identity disturbance. Significant stressors may shake functional identity constitutions and impede personal, social, and role segregation abilities. The COVID-19 pandemic had significant economic impacts [10,11,12] and individual-level consequences [13]. Although social media can have positive outcomes, such as enhancing professional learning [14] and supporting emotional well-being [15], this study focuses on its potential negative effects, particularly during crises.

Unverified messages on the progression of the COVID-19 pandemic and constant updates on deaths, layoffs, and severity further cultivate the strain and exhaustion. The prevailing use of social media, which increases daily across all aspects of life, especially in times where social networking takes precedence over physical gathering, negatively affects mental health and results in buffering stress by assuming maladaptive coping, such as withdrawal and dissociation [16].

Social media usage has a negative effect on mental health [16] and performance [17]. The COVID-19 pandemic negatively affected employees’ well-being, and organizational behavior. Working remotely during the pandemic elevated levels of work stress [18], whereas increased work stress and mental health challenges led to declines in organizational citizenship behavior, indicating that policies regulating work stress and supporting mental health are essential [19]. Contrary to past studies, we explore the impact of anxiety on identity disturbance in a nonclinical setting. We aim to fill the research gap by providing clear evidence that extensive social media exposure during major crises poses significant threats to individuals, with potentially lasting consequences for psychological well-being. In organizational contexts, extensive social media exposure disrupts personal and social domains, leading to conflicts, dissociation, and resource depletion. This study addresses the impact of social media exposure during COVID-19 on mental health outcomes, particularly anxiety and job stress. It seeks to provide insights into the adverse effects of social media exposure on mental health, highlighting the importance of managing social media use and addressing job stress to promote well-being.

## 2. Theoretical Framework

### 2.1. Job Demands–Resources Model

Developed by Demerouti et al. (2001), the job-and-control-support model is a well-established theory that explains how job characteristics influence employees’ psychological well-being [20,21]. The model illustrates how job demands can cause stress, such as heavy workload, role ambiguity, and job-related strain, for employees. However, the model posits that individuals can manage these stressors by utilizing job skills that allow them to gain autonomy and control over their work [22]. Job demands call for the investment of significant physical, cognitive, psychological, and social resources through the ‘employee’ identity role to the detriment of other aspects of the self. Employees who perceive job demands, such as the need for constant availability via social media, as exceeding their resources experience strain, burnout, anxiety, and uncertainty [23]. Not surprisingly, intrapersonal conflict and intrusive negative thoughts and feelings arise, leading individuals to resent the organization or invert harmful debilitating emotions toward themselves [24]. The well-established job demands–resources (JD–R) model provides a useful framework for understanding how job stress induced by a high workload, tight deadlines, and role ambiguity contributes to workplace anxiety.

Certain highly valued resources, such as social support, autonomy in decision-making, and superior feedback, go a long way toward employing adaptive responses when faced with high job demands. As prior work on the subject matter indicates, there is a clear causal interaction between depression, task stress and other social processes. According to this model, increasing job demands, including workload, time pressure, and available cognitive capital, results in job stress. Furthermore, the JD–R model is useful for discerning how job stress underlies the occurrence of anxiety disorders. The model provides grounds for interpreting the role of job demands and resources in shaping psychological well-being. As such, the framework is pragmatic for developing interventions for increasing and preserving psychological resources to reduce workplace anxiety.

### 2.2. The Boundary Theory

The boundary theory developed by Ashfort et al. (2000) and closely related to work–family border theory was founded on the idea that contextual factors can be related to individual outcomes in each domain [25,26]. Since their conception, theories have been widely used in the work–family literature [27,28]. The boundary theory lays the argument for an individual’s internal sense of self that allows for the creation of barriers separating salient self-structural identity from external objects and the environment. Boundaries encompass physical, temporal, and cognitive limitations and are assumed to maintain an integrated, coherent, and stable sense of self [29]. Since its initial development, scholars have used theory to help healthy individuals maintain segregation between diverse segments to facilitate the management of personal, social, and organizational roles [30]. Boundary theory was applied in past studies during the COVID-19 pandemic, indicating that boundary work tactics mitigate burnout among healthcare workers which experienced boundary violations [31]. Moreover, blurred boundaries and a lack of support structures decreased flexibility and affected work–life balance [32].

Any violation or weakening of barriers due to intrinsic conflict or external disruptions leads to disintegration, identity disturbance, inability to commit and, ultimately, anxiety disorders.

### 2.3. Social Media and Anxiety

A myriad of studies have been carried out over the last two years on employees’ social media exposure and usage, yielding opposing results [33]. Understanding the underlying mechanisms leading to psychological distress is key for the successful establishment of optimal procedures, as this approach allows individuals to buffer themselves from stress and reduce anxiety. Employee social media use is negatively associated with both personal and professional segments [9,34]. Social media overload not only creates strain and exhaustion but also depletes individuals of relevant resources, such as time, focus, and attention, creating depletion in the workplace [35]. Moreover, the excessive use of social media has been shown to cause psychological stress [17] and anxiety [36]. Increased usage of social media for work-related purposes during the COVID-19 pandemic and the continuous need for workers to access diverse organizational platforms impose on the personal time of employees thus creating strain [6]. Exhaustion is a common symptom when vast psychological capital must be invested in to remain productive and maintain boundary control [37]. Employees are required to alternate between multiple identities, which further induces anxiety and insecurity [38]. Employees may fear disadvantages, for instance, missing out, falling back on assignments, and failing to pursue collaboration, which places them in a precarious position. Surveys on other demographic factors, such as adults [39,40], have also corroborated the positive association between social media use and depression. According to their findings, social media exposure exacerbates anxiety symptoms through social overload and may result in the development of anxiety disorders [41].

**H1.**  *Social media exposure during COVID-19 pandemic leads to an increase in anxiety.*

### 2.4. Anxiety and Identity Disturbance

Anxiety is commonly characterized as a state manifested in heightened apprehension, feelings of unease, worry, doubt, restlessness, and insomnia [42]. Often, it co-occurs with other mental health conditions and is especially related to depression and identity issues [43]. According to boundary theory, identity development is widely dependent on the constitution of healthy ego boundaries, which serve to maintain the sense of self as distinct from the world, other objects, and individuals. Identity disturbance, by contrast, is characterized by a lack of constancy in sense of self and, by extension, abrupt and dramatic changes in a person’s beliefs, values, aspirations, and behaviors to their detriment [44,45]. A disturbance is accompanied by an underlying sense of insecurity, fear, irritability and, most importantly, role absorption [46]. Considering the substantial role of emotions in the development and sustaining of the coherent ego, and since the social world and the world of the individual are interdependent [47,48], any major disruption in an external environment that disrupts emotional processing can lead to identity disturbance. In the nonclinical population, anxiety propels identity disturbance by destabilizing or diminishing ego boundaries such that individuals feel torn by external stressors or experience gradual disintegration due to internal conflicts [49]. Raemen et al. (2023) found significant indirect effects of identity disturbance on psychological characteristics through anxiety [50]. Moreover, anxiety induces hypersensitivity to interpersonal boundaries, which leads individuals to merge with external objects and other people, i.e., ‘losing oneself in others’. Overall, based on cognizance of boundary theory, anxiety instigates identity disturbance by impeding the formation of healthy ego boundaries, leading to changes in opinion, appraisals, values, and commitment. Work obligations crossing on-the-clock hours through social media during the pandemic, with its ability to erase temporal and physical borders, allow for an intrusion into one’s personal life [51]. This creates an imbalance that threatens healthy ego boundaries, a prerequisite for a strong and grounded sense of identity [52]. Misalignment in work and non-work roles can cause strain, anxiousness, and identity disturbance [52].

Identity disturbance is a prevalent perpetual state of unstable self-image and a dissipating sense of self [53]. Such impairment is especially manifested in the inability to sustain ego constancy and uphold values, objectives, or connections [54]. Identity disturbance and interpersonal dysfunction hinder full identity integration, which allows for normal functioning [55]. Executive functions, i.e., cognitive processing that promotes behaviors leading to goal attainment and self-regulation [56], are related to cognitive flexibility, inhibitory control, and working memory [57]. We contend that all three functions are strongly disrupted in the face of extreme anxiety. Inhibitory control deficits were previously found to be associated with internalizing [58] and externalizing behavioral problems [59]. For instance, anxiety, as an extreme prevalence of negative affect, is, in our understanding, related to identity disturbance [60].

**H2.**  *Anxiety leads to an increase in identity disturbance.*

### 2.5. Job Stress and Anxiety

Job stress is associated with excessive workload [61], intense work demands [62], additional projects [63], changes in organizational structure, and role ambiguity [64,65] and, as such, is one of the most common threats to employee well-being. Job stress poses a significant psychological strain on individuals tasked with balancing multiple roles in and out of the work setting. Strain is a typical consequence of resource depletion and was previously found to be related to mental health impairment, specifically anxiety and depression [62]. Furthermore, considering that social media allows for open accessibility outside of working hours, it has increasingly become a shareholder in this pathological pattern. The ‘always on’ mentality is a source of strain due to employees’ inability to disconnect themselves from organizational obligations, which inevitably leads to anxiety [16]. The JD–R model provides remarkable insight into how increased task demands and lack of available resources can lead to distress. Individuals who are required to assume additional responsibilities but lack sufficient resources, time, capital, or supervisor support to complete these tasks are overwhelmed and excessively self-aware, distraught, drained and unconfident, which may lead to anxiety.

**H3.**  *Job stress leads to an increase in anxiety.*

### 2.6. Moderating and Mediating Effects

Social media usage was previously found to be associated with higher levels of job stress and burnout [23,66,67]. When overwhelming and prevailing notifications, updates, and news about social media streams accumulate, they become an underlying cause of distraction and interference [68], resulting in poor work performance, disorganized behavior, and increased stress levels [69,70].

Social media usage was previously found to have a stressful effect on individuals [71]. On the one hand, it cultivates the effect of constant connectedness, which can be a source of both positive sensibility [72] and strain [73]. Some scholars have found empirical evidence that social media usage across industries during working hours is associated with negative experiences, such as strain, distraction, reduced productivity, and increased work demand, which leads to an increase in ambiguity, imbalance, and anxiety [74]. However, the relationship between social media and anxiety is complex, and recent studies have proposed that job stress moderates this relationship [75]. The moderating effect can be attributed to individuals high in work stress being more prone to the negative outcomes of social media usage due to a lack of resources for coping with the additional stressors caused by social media. In contrast, employees scoring lower on work stress may be more resilient to the detrimental consequences of social media due to excess resources for adaptive coping.

**H4.**  *Job stress moderates the relationship between social media exposure and anxiety.*

According to the boundary theory [32], when physical, temporal, and cognitive domain-defining limitations are misaligned or breached, there are severe consequences for an individual’s psychological well-being. According to Kreiner, Hollensbe, and Sheep (2006), work and non-work domains are two distinctive and often incompatible areas segregated by boundaries pragmatically posed by employees to facilitate navigating through different identity roles [31,36]. According to Kossek et al. (2012), who developed a measure of boundary management, there are three subscales of stress-inducing behaviors, namely cross-role interruption behaviors, such as work interruption non-work behaviors and non-work interruption work behaviors, boundary control, and work and family identities [76]. To buffer from work stress and maintain balance, individuals rationally allocate resources, assuming the appropriate worldviews, emotions, and behaviors at home and in the work setting [77]. However, the explosive nature of interactions via social media interweaves multiple life domains and transcends professional and personal boundaries, creating conflict among multiple identities [5]. As previously mentioned in the characterization of identity, alters can presuppose the positions characterized by clear responsibilities and behavioral expectations. When roles are incongruent, inevitably, there is a collision of one meaning against a certain alternative position [78,79]. The collision of two disparate identities in a single situation leads to the sustaining of one activity with the detriment of the other. Deflection leads to the psychological appraisal of the world as uncertain and unpredictable, especially when the narrative is viewed from two or more differing perspectives, resulting in severe distress and anxiety. Fragmentation under significant pressure leads to hopelessness, lack of control and, ultimately, loss of identity. Dissociation is often the maladaptive mechanism individuals use to overcome trauma and distance themselves from anxiety [80].

**H5.**  *The relationship between social media exposure and identity disturbance in individuals is mediated by anxiety.*

**Figure 1:**
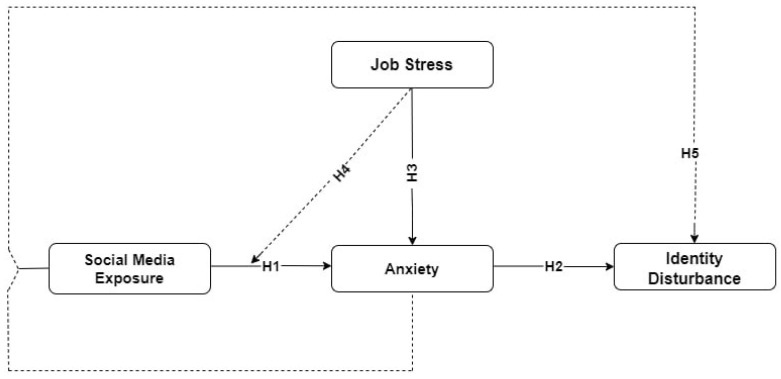
Conceptual framework.

The research model is depicted in Figure 1.

## 3. Materials and Methods

### 3.1. Data Collection

The survey strategy was employed, and the cross-sectional study was executed. The data collection was conducted in the USA during a three-month interval after the second wave of the COVID-19 pandemic. To target the population, questionnaires were administered online using Survey Monkey. Self-reported measures were used to assess the variables of interest. Likert scales were used to collect data on the level of job stress, identity disturbance, and social media exposure during the pandemic. Links were sent via e-mail to subjects through official company communication channels. All participants received detailed instructions on the survey and confidentiality assurance. Prior to engaging in the survey, informed consent was obtained in the form of explicit acknowledgment of the understanding on study objectives and their rights. To ensure conformity with ethical principles involving human subjects, the research adhered to the Helsinki Declaration. The study gathered demographic data from 282 individuals in the USA during the COVID-19 pandemic. The majority of the participants were male (63.1%), with 36.9% female. In terms of participant age, the largest group included those older than 60 years (51.4%), followed by those aged 50–59 years (21.6%), 40–49 years (12.8%), 30–39 years (11.3%), and 21–29 years (2.8%). The highest proportion of respondents held a PhD or higher (65.2%), followed by a postgraduate degree (29.4%), a bachelor’s degree (4.3%), some college but no degree (0.4%), and a high school degree or equivalent (0.7%).

### 3.2. Measurements

#### 3.2.1. Job Stress

Job stress levels were measured using a 5-item scale adapted from Maunder et al. (2006), showing a Cronbach’s alpha of 0.884 thus indicating good reliability [81]. The participants were asked to rate their experiences on a 5-point Likert scale ranging from 1 (not at all) to 5 (extremely). Sample items included “I felt more stressed at work” and “I had to work that normally I don’t do”. 

#### 3.2.2. Identity Disturbance

Identity disturbance was assessed using the Personality Structure Questionnaire (PSQ) created by Pollock et al. (2001); this scale consisted of 16 items, with 8 items reverse coded, and achieved a Cronbach’s alpha of 0.865 [82]. Sample items included “I am split between two (or more) ways of being, sharply differentiated from each other” and “My mood can change abruptly in ways which make me feel unreal or out of control”.

#### 3.2.3. Social Media Exposure

To assess social media exposure during the pandemic, a social media exposure scale adapted from Ng et al. (2018), with a high reliability (Cronbach’s alpha of 0.95), was utilized [83]. Participants rated each item on a five-point Likert scale from “Strongly disagree” (1) to “Strongly agree” (5). Sample items were “Many people on my online social network frequently posted status updates about COVID-19 on their Facebook timeline, Twitter feed, etc.” and “I saw many posts that relate to health information about COVID-19 that were shared by people in my social network”.

#### 3.2.4. General Anxiety

General anxiety symptoms were measured using the seven-item Generalized Anxiety Disorders (GAD-7) scale, which has demonstrated sufficient reliability in past research, achieving a Cronbach’s alpha of 0.944. The scale included seven items rated on a scale ranging from “Not at all” (1) to “Nearly every day” (5). Sample items included “Becoming easily annoyed or irritable” and “Feeling afraid as if something awful might happen”.

### 3.3. Statistical Analysis

The statistical analysis was conducted using SPSS Amos 29 software. Harman’s single-factor methodology was applied addressing common method bias. Confirmatory factor analysis (CFA) was performed to test the measurement model, using fit indices including the chi-squared to degrees of freedom ratio, Root Mean Square Error of Approximation (RMSEA), Standardized Root Mean Square Residual (SRMR), Normed Fit Index (NFI), Incremental Fit Index (IFI), Tucker–Lewis Index (TLI), and Comparative Fit Index (CFI) to evaluate the fit of the model. Reliability and validity check of the measurement tools were performed to ensure their accuracy and that they measured what they intended to. Cronbach’s alpha and Composite Reliability (CR) for internal consistency and Average Variance Extracted (AVE) for convergent validity were calculated. This study utilized a two-step SEM approach consisting of both measurement model and structural model testing. Relationships between the model variables of social media exposure, identity disturbance, anxiety, and job stress were analyzed using standardized beta coefficients, standard errors, t-values, and *p*-values.

## 4. Results

Harman’s single-factor methodology was employed to test for common method bias. The variance retrieved using a single component was 7.571%, which was less than 50%, indicating that no common method bias existed in this study [84].

**Figure 2:**
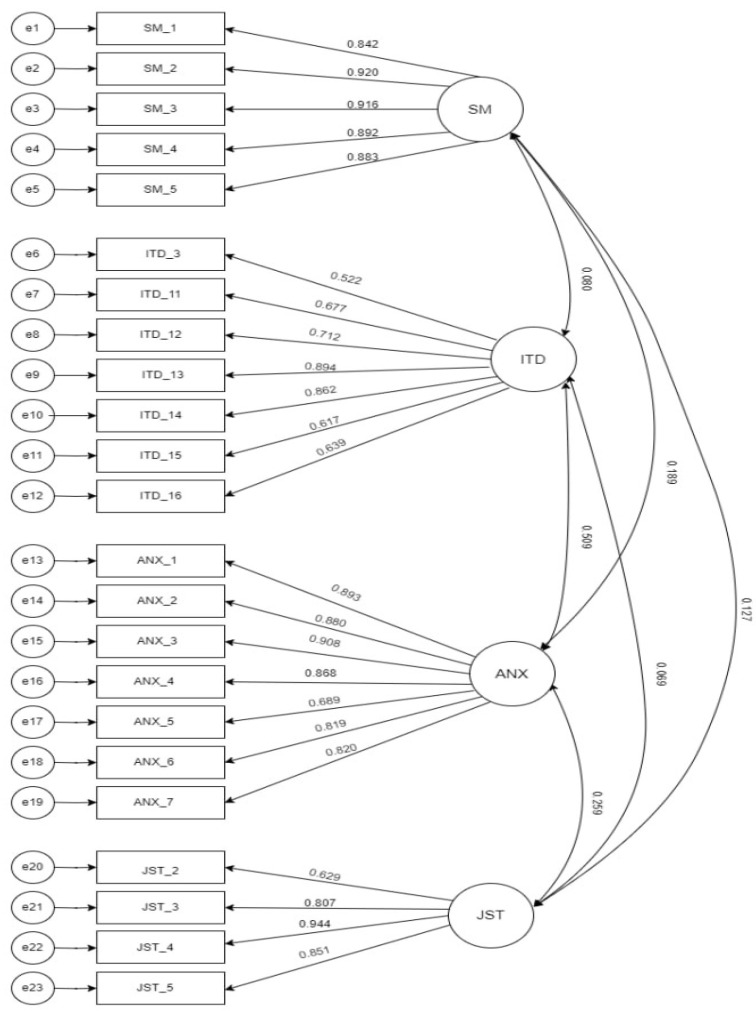
Graphical Representation of measurement model.

Confirmatory factor analysis (CFA) was performed to test the measurement model. The CFA is illustrated in Figure 2. The chi-squared value was 532.567 with 224 degrees of freedom, resulting in a chi-squared to degrees of freedom ratio of 2.378, indicating good model fit. The root mean square error of approximation (RMSEA) was 0.070, which was below the recommended threshold of 0.08, and the standardized root mean square residual (SRMR) was 0.061, which was also below the recommended threshold. The normed fit index (NFI) was 0.899, the incremental fit index (IFI) was 0.939, the Tucker–Lewis index (TLI) was 0.931, and the comparative fit index (CFI) was 0.939, all of which indicated good model fit (Table 1).

Table 2 displays the reliability and validity analysis of the constructs. Cronbach’s alpha and composite reliability (CR) were used to assess internal consistency, with alpha coefficients of 0.95, 0.865, 0.944, and 0.884 for social media exposure, identity disturbance, anxiety, and job stress, respectively. All the constructs had composite reliability values above 0.7, indicating high reliability. Validity was assessed using the average variance extracted (AVE) and factor loading, with all the constructs having AVE values of above 0.5, indicating good convergent validity, and factor loadings of above 0.7, indicating good discriminant validity.

The variance inflation factor (VIF) values indicate that multicollinearity is not a concern in this study (Table 3).

The bootstrap procedure was used with the recommended 5000 subsample size for hypothesis testing. The direct effects of the hypotheses were examined.

The direct relationships between the variables and the standardized beta coefficients, standard errors, t values, and *p* values are displayed in Table 4. The results indicate that social media exposure (SM) has a significant positive effect on anxiety (ANX) (β = 0.173, *p* = 0.007), anxiety has a significant positive effect on identity disturbance (ITD) (β = 0.542, *p* = 0.000), and job stress (JST) has a significant positive effect on anxiety (β = 0.232, *p* = 0.001). Furthermore, the results indicate that anxiety mediates the relationship between social media exposure and identity disturbance (β = 0.094, *p* = 0.004). Consequently, the hypotheses H1 (SM → ANX), H2 (ANX → ITD), H3 (JST → ANX) are all accepted.

**Figure 3:**
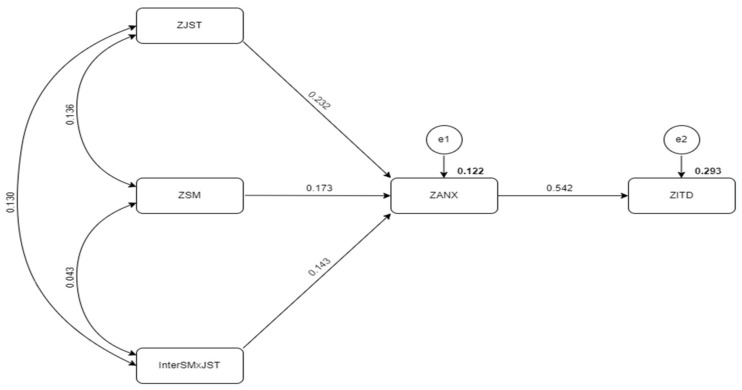
Graphical representation of structural model.

The results also support H4 (β = 0.143, *p* < 0.001), where job stress moderates the relationship between social media exposure and anxiety (Figure 3). This means that the effect of social media exposure on anxiety is stronger for employees experiencing high levels of job stress. Overall, these findings suggest that job stress exacerbates the negative impact of social media exposure on mental health outcomes, highlighting the importance of addressing both factors in promoting employee well-being. The results are presented in Table 5. The H5 hypotheses testing for the indirect effect showed a significant indirect effect (β = 0.094, *p* < 0.005).

The analysis of the data revealed associations between the measured variables, providing insights into the relationships among job stress, anxiety, identity disturbance, and social media exposure during the COVID-19 pandemic. The results indicate that higher levels of pandemic-related stressors were associated with increased job stress and identity disturbance. Additionally, social media exposure during the pandemic was found to be a contributing factor to increased anxiety and worry among participants.

## 5. Discussion

The widespread use of social media has transformed the way individuals and organizations communicate, creating new channels for engagement with employees and clients in the face of adversity and lockdown measures. Despite the potential benefits, however, the impact of social media exposure and usage on both individual and organizational outcomes remains a topic of ongoing research, especially in light of the recent COVID-19 crisis. In particular, the digital transformation and introduction of collaborator platforms and organizational social networks has led to a high level of job stress, with individuals expected to be available at all hours and manage multiple roles simultaneously. This stress can lead to burnout, emotional distress, and difficulty maintaining healthy ego boundaries, which are critical for a strong and stable sense of identity. In this context, the well-established job-and-resources (JD–R) model and boundary theory provide useful frameworks for understanding the complex interplays among job demands, workplace anxiety, and the effects of social media usage on individual well-being.

Hypothesis 1, proposing that social media exposure leads to anxiety, was accepted. By synthesizing cognizance from the extensive literature on the subject, we theorized that anxiety arises when a seemingly endless stream of content, updates, notifications, and messages related to COVID-19 leaves workers vulnerable to strain. Our findings corroborate those of Wu et al. (2021), Cao and Yu (2019), and Sun et al. (2021) [17,36,92]. As employers increasingly blur the boundaries between professional and personal time of employees the question of ignoring the influx of information becomes less of a choice for employees.

Hypothesis 2, stating that anxiety has a positive effect on identity disturbance, was confirmed by empirical analysis and thus accepted. Furthermore, in line with Livesley’s (2003) arguments, we propose that when there are disturbances in one’s sense of identity and interpersonal relationships, as in the case when remote working and social media is imposed on employees, it can impede the integration of one’s identity, which, in turn, affects one’s ability to function normally [55]. Executive functions, which involve cognitive processes that help individuals achieve goals and self-regulate, such as cognitive flexibility, inhibitory control, and working memory [56,57], are all seriously impaired in situations of extreme anxiety during adversity. For example, anxiety, which is characterized by a pervasive sense of negative affect, is linked to disturbances in one’s sense of identity [60]. This finding is in line with the arguments of Obrenovic et al. (2020) and Raemen et al. (2023) [49,50].

Hypothesis 3, which proposes that job stress leads to anxiety, was confirmed. Job stress is a common phenomenon in modern workplaces and can significantly impact employees’ mental health. Anxiety is one of the most prevalent mental health disorders, and job stress has been shown to be a contributing factor to its development. Prior research indicates that job stressors such as workload, role ambiguity due to changes in the organizational setting, job insecurity in the face of COVID-19, and the lack of control can lead to increased anxiety among employees [21]. Additionally, a lack of support from supervisors and colleagues can exacerbate anxiety symptoms, creating a vicious cycle of job stress and mental health issues.

Hypothesis 4, stating job stress moderates the relationship between social media exposure and anxiety, was accepted. Individuals who experience high levels of job stress may be more susceptible to the negative effects of social media exposure on anxiety. Understanding this phenomenon is essential for developing effective interventions and treatments for individuals who may experience anxiety related to social media use. The relationship between social media exposure and anxiety has been extensively studied in recent years [16,17], showing a positive link between the two variables [93], whereas job stress is a well-known predictor of anxiety and has been found to have a negative impact on mental health [94,95].

Finally, we found support for the proposition that anxiety mediates the relationship between social media exposure and identity disturbance (H5). Our findings suggest that anxiety may be a critical mechanism through which social media exposure contributes to identity disturbance in individuals. Therefore, interventions that target anxiety management may be an effective way to prevent or reduce identity disturbance among individuals who frequently use social media. The results of the current study support the claims that social media exposure and job stress lead to anxiety and that anxiety has a positive effect on identity disturbance. Additionally, we found evidence for both moderating and mediating effects.

The findings of the study have relevant implications for individuals, organizations, policymakers, and mental health professionals. We lay the groundwork for the development of new organizational policies and more efficient processes tailored to buffer stress, strain, and work–family conflict. Given that employee creativity can be enhanced through various means [96] and that even pressure to perform can lead to boundary-spanning behavior [97] and innovation [98], organizations should find ways to manage stress into a positive outcome. For instance, coping styles can be useful in alleviating distress [99], while spiritual leadership [100] and mindfulness practices can mitigate the adverse effects of emotional exhaustion and enhance psychological resilience [101].

## 6. Conclusions

The study has laid the groundwork for explaining how job stress moderates the relationship between social media exposure and psychological impairment. It provided evidence for the mediating effect of anxiety on the relationship between social media exposure during the COVID-19 pandemic and identity disturbance thus filling key research gaps. Previous studies have examined the effect of identity disturbance on anxiety, especially related to PDs in clinical populations, limiting the generalizability of the findings. Conversely, this study explored the inverse relationship, namely how anxiety can lead to identity disturbance in a nonclinical setting thus broadening the understanding of the relationship between the two constructs. This is an important contribution, as it sheds light on a less-explored direction of the relationship between anxiety and identity. By applying the job demands–resources (JD–R) model and boundary theory, we offered a comprehensive understanding of these effects, underscoring the importance of managing social media use and addressing job stress to promote well-being.

The findings highlight the need to address anxiety symptoms to prevent and treat identity disturbance. Additionally, this initial work can help organizations develop policies and programs to mitigate the negative impacts of social media exposure on mental health, especially for individuals experiencing job stress. Furthermore, these conclusions can help individuals better understand the potential risks associated with social media use and make more informed decisions about their social media habits.

### Limitations and Future Studies

The study has limitations as it is of a cross-sectional design, with data being collected once during the pandemic. Consequently, longitudinal studies can be performed in the future to better understand the relationships over time. The study relies on self-reported data, which can introduce biases. Additionally, there are threats to generalizability given that the study was performed in a pandemic context and as the highly educated sample may not be representative of the larger population. Thus, future research should investigate more heterogeneous samples in a post-pandemic context. Further research is needed to better understand the complex interplays among social media exposure, anxiety, and identity disturbance, as well as to develop effective interventions to mitigate the negative impact of social media on mental health. In particular, future studies could empirically explore boundary theory and how it impacts the interplay between social media exposure and job stress, investigating mechanisms such as boundary permeability and flexibility rather than only using it as a contextual theory for identity disturbance. Finally, the research model is constrained to only four variables. Future research can test the impact of individual differences, such as mental health history and personality traits, in relation to anxiety and identity disturbance. The use of novel technology such as generative AI in the workplace and in private can also be investigated as potential triggers of job stress.

## Figures and Tables

**Table 1:** Model fit Indices.

Fit Index	Cited	Fit Criteria	Results	Fit (Yes/No)
X2			532.567	
DF			224	
X2/DF	[85]	1.00–5.00	2.378	Yes
RMSEA	[86]	<0.08	0.070	Yes
SRMR	[87]	<0.08	0.061	Yes
NFI	[88]	>0.80	0.899	Yes
IFI	[89]	>0.90	0.939	Yes
TLI	[90]	>0.90	0.931	Yes
CFI	[91]	>0.90	0.939	Yes

**Table 2:** Reliability and Validity Analysis.

Alpha, Composite Reliability and Validity Analysis					
Construct	Items	Loading	Alpha	CR	AVE
			>0.7	>0.7	>0.5
Social media exposure	SM_1	0.842 ***	0.95	0.95	0.793
	SM_2	0.920 ***			
	SM_3	0.916 ***			
	SM_4	0.892 ***			
	SM_5	0.883 ***			
Identity disturbance	ITD_3	0.522 ***	0.865	0.876	0.51
	ITD_11	0.677 ***			
	ITD_12	0.712 ***			
	ITD_13	0.894 ***			
	ITD_14	0.862 ***			
	ITD_15	0.617 ***			
	ITD_16	0.639 ***			
Anxiety	ANX_1	0.893 ***	0.944	0.944	0.709
	ANX_2	0.880 ***			
	ANX_3	0.908 ***			
	ANX_4	0.868 ***			
	ANX_5	0.689 ***			
	ANX_6	0.819 ***			
	ANX_7	0.820 ***			
Job stress	JST_2	0.629 ***	0.884	0.886	0.666
	JST_3	0.807 ***			
	JST_4	0.944 ***			
	JST_5	0.851 ***			

* Indicates significant paths: *** *p* < 0.001.

**Table 3:** VIF/Multicollinearity.

Constructs	1	2	3	4
1. Anxiety		1.113	1.534	1.464
2. Identity disturbance	1.012		1.427	1.416
3. Social media exposure	1.024	1.049		1.042
4. Job stress	1.023	1.089	1.09	

**Table 4:** Hypotheses testing.

Hypothesis	Indirect	Std.	Std.	T	*p*
	Relationships	Beta	Error	Value	Values
H1	SM → ANX	0.173	0.057	3.035	0.007
H2	ANX → ITD	0.542	0.052	10.423	0.000
H3	JST → ANX	0.232	0.054	4.296	0.001

**Table 5:** Moderating Effect.

Hypothesis	Direct	Std.	Std.
	Relationships	Beta	Error
H4	InterSMxJST	0.143 ***	0.059

* Indicates significant paths: *** *p* < 0.001.

## Data Availability

Dataset available on request from the authors.
